# Strong magnetic field-assisted growth of carbon nanofibers and its microstructural transformation mechanism

**DOI:** 10.1038/srep09062

**Published:** 2015-03-12

**Authors:** Chengzhi Luo, Qiang Fu, Chunxu Pan

**Affiliations:** 1 School of Physics and Technology, and MOE Key Laboratory of Artificial Micro- and Nano-structures, Wuhan University, Wuhan 430072, China; 2 Center for Electron Microscopy, Wuhan University, Wuhan 430072, China

## Abstract

It is well-known that electric and magnetic fields can control the growth direction, morphology and microstructure of one-dimensional carbon nanomaterials (1-DCNMs), which plays a key role for its potential applications in micro-nano-electrics and devices. In this paper, we introduce a novel process for controlling growth of carbon nanofibers (CNFs) with assistance of a strong magnetic field (up to 0.5 T in the center) in a chemical vapor deposition (CVD) system. The results reveal that: 1) The CNFs get bundled when grown in the presence of a strong magnetic field and slightly get aligned parallel to the direction of the magnetic field; 2) The CNFs diameter become narrowed and homogenized with increase of the magnetic field; 3) With the increase of the magnetic field, the microstructure of CNFs is gradually changed, i.e., the strong magnetic field makes the disordered “solid-cored” CNFs transform into a kind of bamboo-liked carbon nanotubes; 4) We propose a mechanism that the reason for these variations and transformation is due to diamagnetic property of carbon atoms, so that it has direction selectivity in the precipitation process.

In general, one-dimensional carbon nanomaterials (1-DCNMs) including carbon nanotubes (CNTs) and carbon nanofibers (CNFs) exhibit various remarkable properties, which are attractive for wide potential applications in micro-nano-electrics and devices^1^,^2^. However, its applications virtually depend upon the growth controllability in direction, morphology and microstructure, etc.^3^,^4^.

It has been well-known that applied electric and magnetic fields exhibit effective controllability on the growth direction, morphology and microstructure of 1-DCNMs. The electric field has received the most attentions^5^,^6^. It was reported that the focusing electric field exhibited a distinct influence on purity, morphology and yield of the arc-generated CNTs^7^,^8^. In our previous work, it was found that an additional electric field could not only align the CNTs growth along the electrostatic force, but also improved the diameter uniformity and the crystallinity of the graphite sheets^3^,^9^. Compared to electric field, few papers focused upon magnetic field for controlling growth of 1-DCNMs. This is because that magnetic field is more difficult to be introduced and controlled in the preparation process. Generally, there are three strategies to introduce a magnetic field: pre-synthesis, during-synthesis and post-synthesis. The pre-synthesis introduction is a process to control the CNTs growth direction via controlling the catalyst deposition direction by using a magnetic field^10^. The post-synthesis introduction is based on the radial and transverse magnetic anisotropy of CNTs or the decoration of CNTs by magnetite nanoparticles and their orientation in a magnetic field^11^.

In fact, the most effective strategy to control the growth direction of 1-DCNMs is to introduce a magnetic field during the preparation. For example, it has been reported that magnetic field provided an advantageous tool to improve the purity and productivity of CNTs in an electric arc discharge method^12^,^13^,^14^. G. Xing et al.^15^ introduced an alternating transverse magnetic field (0.8 mT, 5 Hz) to arc discharge in liquid (de-ionized water and liquid nitrogen), and arc plasma swung periodically with the effect of Lorentz force. For arc discharge in water, curled CNTs and few-layer nano-structures were found in the products, and for arc discharge in liquid nitrogen, twists of single-walled CNTs appeared in the products. D. Wei et al.^16^ developed a magnetism-assisted chemical vapor deposition(CVD) process by using a Sm_2_Co_17_ magnet (0.2 T, 50 mT/cm gradient around the surface of the magnet). They found that more branched CNTs were obtained when the magnetic field was vertical to the CNTs growth direction, while higher contents of Fe-encapsulated CNTs were found in case of the magnetic field was parallel to the CNTs growth direction. In addition, N. Ohmae^17^ attempted to get a curved CNTs in a plasma-enhanced CVD (PECVD) system by applying an external Nb-Fe-B magnet (center magnetic field strength 0.01 T). It was observed that by changing the direction of the line of magnetic force during preparation, the arch-shaped structure of CNT resulted, which was attributed to the force acting on Fe nanoparticles.

In our previous work^18^, a flame method was used for growing CNTs. The neodymium-iron-boron (Nd-Fe-B) magnet (surface magnetic strength 0.38 T) was applied as an external magnetic field around the flame. It was found that not only the well-aligned CNTs grew along the magnetic field due to the CNT's diamagnetic property, but also the crystallinity of CNTs graphite sheets was improved. The calculation revealed that the magnetic force acting upon the CNT itself was much larger than that upon the catalyst particle at the tip. Up to now, the growth mechanism of CNTs in a magnetic field has been clearly understood, but only a small magnetic field (less than 0.01 T in the center) was used.

In addition, in 1-DCNMs family, there is a kind of “solid-cored” carbon nanofibers with amorphous structure^19^, which exhibited special advantages in microwave-absorbing materials and heteroarchitectured photocatalytic composites^20^,^21^. The alignment and microstructure of CNFs are essential for the realization of practical devices. Researchers agree that CNFs are aligned due to the presence of the electric field in a PECVD process^22^. M. Meyyappan et al. prepared CNFs by combining hot filament with a direct current plasma approach. They demonstrated that the filament wire was important primarily in the improvement of CNF growth quality^23^. Both theoretical calculations and experimental observations have revealed that the internal structure of the vertically aligned CNFs grown by catalytic PECVD could be influenced by the crystallographic orientation, structure and shape of the catalytic nanoparticles^24^,^25^. In our previous work^26^, we introduced an electric field (0–50 V/mm) in a CVD system, and found that an increase in electric field produced a smaller diameter and narrower diameter distribution of CNFs. The calculation revealed that the mechanism for diameter change was due to two reasons, i.e., Ni catalyst particles became liquid at the reaction temperature, and the diameter of the Ni catalyst became smaller under the electric field. But we did not observe the change of the CNFs growth direction.

In this paper, we introduced a strong magnetic field (up 0.5 T at the center magnetic field) to study its effects on the CNFs growth and microstructural transformation. The experimental setup and schematic diagram were shown in Figure 1. It was found that a strong magnetic field could not only control the CNFs growth direction, but also improve the diameter uniformity and microstructural transformation, i.e., the microstructure transformed from the disordered “solid-cored” CNFs into the bamboo-liked CNTs. This phenomenon will exhibit a great significance for its further applications.

Figure 2 shows SEM morphologies of CNFs that were prepared without magnetic field. Obviously, CNFs were in a random and entangled orientation, and the diameter was about 50 nm. Figure 3 shows SEM morphologies of the CNFs under different magnetic field strengthes. It revealed that when magnetic field 0.1 T was added, the CNFs started to grow perpendicular to the substrate and slightly became aligned parallel to the direction of magnetic field. In addition, with the increase of magnetic field strength, the alignment of CNFs were further improved. More importantly, a strong magnetic field greatly improved the controllability and repeatability of the growth of the aligned CNFs.

In order to further verify the influence of magnetic field on the growth of aligned CNFs, we conducted some control experiments. First, we designed an experiment to prepare CNFs with a magnetic field 0.5 T at the first 5 min, and then withdraw the magnetic field at the last 5 min, as shwon in Figure 4a and 4b. Clearly, from the side view, the CNFs could be divided into two distinct portions, i.e., the lower portion exhibited a dense and aligned CNFs with small diameter, which was similar to the CNFs prepared under the magnetic field, while the upper portion was the random and entangled CNFs with large diameter. This meant that in the present experiment the dense enough CNFs could not grow aligned without magnetic field. Second, we just grew a bundle of CNFs and changed the magnetic field direction parallel to the substrate. In this situation, the CNFs were not dense enough. If the alignment mechanism was “crowding effect”, the CNFs would grow in a random orientation (as shown in Figure 4c). The morphology of the CNFs in Figure 4d changed obviously when comparing to Figure 4c. The CNFs grew in a random orientation in Figure 4c, while the CNFs grew in a certain orientation in Figure 4d. That meant the magnetic field controlled the CNFs orientation. This experiment verified that the alignment mechanism was the “magnetic field effect” rather than the “crowding effect”. The similar experiments were also reported by Nobuo Ohmae^17^.

In general, the force for a magnetism-assisted growth of well-aligned CNTs was mainly from the effect of magnetic field upon CNT itself, and the force acting upon the catalyst particles was too weak and could be neglected^18^. Here, we propose a model to explain the growth mechanism of the aligned CNFs in a strong magnetic field based on the following two reasons, as shown in Figure 5.

1) The mechanism of the catalytic growth of CNFs has been studied over a long period of time. Although consensus has been reached with respect to the different growth steps, still uncertainties exist about some details. In the present case, the carbon source was C_2_H_2_, and the catalyst was Ni. Formation of CNFs by Ni catalyst could be explained as follows: (1) The first step was the decomposition of C_2_H_2_ on the Ni surface. That is, carbon atoms deposited on the surface with the concomitant release of gaseous products like molecular hydrogen and carbon dioxide. (2) In the second step, the carbon atoms dissolved in and diffused through the bulk of the Ni particle, although some contribution of surface diffusion could not be excluded. (3) The final step was the precipitation of the carbon in the form of a CNF consisting of graphite at the other side of the Ni particle^19^. If the axis of a CNF grew perpendicularly to the substrate and coincided with the direction of the magnetic field, the gradient of magnetic field would repell carbon atoms and made the atoms precipitate uniformly on both side of the bottom of the catalyst particle/nanofiber interface. Therefore, the CNFs continued to grow vertically, as shown in Figure 5a. However, if there were a spatial fluctuation in the carbon precipitation at the interface, the growth of the CNFs would deviate from vertical alignment. In this case, carbon atoms would still be repelled by the repulsive force and precipitate preferential on the bottom side of the catalyst particle/nanofiber interface. At last, the preferential carbon precipitation led to the bottom side of the catalyst particle/nanofiber grow faster, which resulted in the well- aligned CNFs grew along the magnetic field line, as shown in Figure 5b.

2) Because catalyst Ni particle generally lost its ferromagnetism and transformed into paramagnetism, when the temperature increased above its Curie point of 358°C^18^. The strong magnetic force pulled the Ni particles along the magnetic field line and also controlled the growth direction of CNFs^17^.

Figure 6 illustrates Raman spectra of the CNFs under different magnetic field strengthes. Obviously, the intensity of two main D and G peaks was changed with increasing of the magnetic field strength. It is well-known that D peak at 1354 cm^−1^ was due to the presence of amorphous carbon and defects, and G peak at 1590 cm^−1^ was from C-C band longitudinal vibrations along the graphite lattice for characterizing the graphitizing quality. The integrated intensity ratio I_D_/I_G_ for D band and G band is widely used for evaluating the graphitizing quality in graphitic materials^27^. The experimental results revealed that the integrated intensity of disordered D band and the I_D_/I_G_ ratio became smaller and smaller with increasing of the magnetic field strength, that was, from 2.54 at 0 T decreased into 0.82 at 0.5 T, as shown in Figure 6b. It indicated that the graphite layers changed when a strong magnetic field was applied, that was to say, a strong magnetic field induced a microstructural transformation from the disorder “solid-cored” CNFs into a kind of graphitized structure.

Figure 7 shows HRTEM micrographs of the CNFs prepared under 0 T and 0.5 T, respectively. Obviously, the CNFs were a kind of “solid-cored” fiber with a disordered structure and 50 nm in diameter, when no magnetic field was applied. However, under 0.5 T magnetic field, the diameter of CNFs became smaller and more uniform around 20 nm. Except the narrower diameter, the fibers have been transformed into a kind of bamboo-liked CNTs with a graphitized structure. Furthermore, it was notable that after applying a strong magnetic field, Ni particles changed into a cone shape, i.e., large in the top and small at the bottom. We believed that the shape transformation of Ni catalyst played a key role on the CNFs' diameter narrowing and microstructural transformation. In order to accurately measure the diameter of the catalyst particles, we randomly took HRTEM images up to 100 photos for each sample and got statistical values, as shown in Figure 7e. The diameter of the catalyst particles was decreased with the increase of magnetic field. The narrowness of the Ni catalyst particles should be responsible for the changes of the CNFs diameters.

Over the past decades, the bamboo-liked CNTs have been obtained by many methods, such as microwave plasma enhanced CVD^28^, thermal CVD^29^, plasma enhanced CVD^30^. The real-time TEM imaging revealed that the shape of Ni catalyst particle changed constantly during the growth of the bamboo-liked CNTs, and the bamboo-liked CNT growth followed the adsorption-decomposition-surface diffusion-step nucleation process^31^. Different from previous studies, the present bamboo-liked tubes were transformed from the disorder “solid-cored” CNFs with assistance of a strong magnetic field, which has not been reported. Therefore, we modified the mechanism based on the previous research^32^ to illuminate the microstructural transformation of the CNFs, as shown in Figure 8.

1) When a strong magnetic field was applied during growth process of the CNFs, the local magnetic field strength surrounding the catalyst particles would be enhanced^18^. Due to diamagnetism of carbon atoms, the magnetic torque of carbon atoms was opposite to the magnetic field line, which created a repulsive force on it and the carbon atoms intended to precipitate along the opposite direction of the strong magnetic field line^33^, i.e., precipitated along the both sides of the bottom of the catalyst particle/nanofiber interface. Therefore, a compressive force formed at the bottom of the particles, which led to the molten catalyst particle becoming a cone shape, as shown in Figure 7d. The CNFs' diameter was determined by the size of catalyst particles^26^, which was decreased with increase of the magnetic field. Therefore, the diameter of CNFs was reduced with increase of the magnetic field.

2) The degree of order and arrangement of graphite layers could be influenced by the surface property of catalysts. It is well-known that the carbon diffusion in catalyst particle depends upon its stress and deformation states. The preferential precipitation of carbon atoms induces deformation for the particle, which results in the nucleated graphite layers parallel to the surface lattice of catalyst particle^3^. In the present case, these paralleled graphite layers formed an ordered structure with less vacancy and defect. And therefore, with the growth of the bamboo-liked CNTs, more and more parts of the catalyst particle were sucked into the tube, meanwhile the surface area explored in C_2_H_2_ atmosphere was decreased, which meant that the transportation of carbon through the catalyst particle gradually decreased. The catalyst particle was stretched to a reverse cone shape, because of the preferential precipitation of carbon atoms. When a compressive force from the preferential precipitation of carbon atoms decreased to such an extent that smaller than the surface tension of the catalyst particle, the portion of the sucked and stretched catalyst would be pulled back under the combined action of the surface tension of the particle and stress of the tube. In this way, a piece of bamboo was formed, and a new circle would start at the lower part of the catalyst particle, and produced another piece of bamboo, as shown in Figure 8. If the stretched part of a particle could not be completely pulled back, a droplet of the catalyst particle would be kept in the compartment of the tube, as shown in the yellow box of Figure 8c.

In summary, we developed a facile and effective process to have a controllable preparation of CNFs by applying a strong magnetic field in a CVD system. The experimental results revealed that an additional strong magnetic field could not only control the CNFs growth direction (along the magnetic field line) but also improved the diameter uniformity and change the microstructure of the CNFs. In other words, a strong magnetic field could make the disorder “solid-cored” CNFs transform into the bamboo-liked CNTs. Our experimental results provide a new route to synthesize “graphite-amorphous” carbon heterojunction and isomeric “graphite-nongraphite-graphite-nongraphite” CNTs and would have wide potential applications in micro-nano-electrics and devices.

## Methods

**Figure 1:**
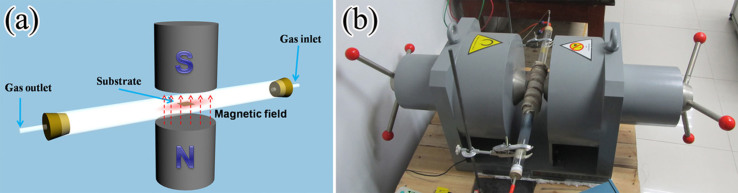
Experimental setup. (a) schematic diagram; (b) factual system.

Figure 1 shows the experimental setup and schematic diagram for preparing the CNFs within a magnetic field. The inner diameter of quartz tube in the self-made CVD furnace was 30 mm. The electromagnet (Litian Magnetoelectrican Science & Technology Co. Ltd, China) with a magnetic gap of 70 mm provided the magnetic field. Its center magnetic field strength was continuously adjustable from 0 to 0.5 T. The experimental details were as follows: 1) A nickel (Ni) nanocrystalline layer was electrodeposited on the copper substrate. The electrolyte and parameters have been described elsewhere^18^; 2) The substrate was placed in the quartz tube reactor and 200 sccm Ar was continuously introduced into the tube; 3) Heating the reactor to the temperature 700°C, and when the temperature reached the desired value, a strong magnetic field strength involving 0 T, 0.1 T, 0.2 T, 0.3 T, 0.4 T and 0.5 T was perpendicularly added on the react zone; 4) The CNFs grew on the substrate by adding 10 sccm C_2_H_2_ at 700°C; 5) After 10 min, closed C_2_H_2 _gas and cooled the substrate to room temperature in Ar gas protection.

The morphology and microstructures of the samples were characterized using a scanning electron microscope (SEM) (S-4800, HITACHI, Japan) and high resolution transmission electron microscope (HRTEM) (JEM 2010FEFHRTEM, JEOL, Japan). Raman spectroscopy (Join Yvon LabRam HR, HORIBA, France) was used to characterize the graphitizing quality and the order degree of the graphite sheets. The power of laser was 10 mW, and the laser excitation was 488 nm.

## Author Contributions

C.L., Q.F. and C.P. conceived and designed the project. C.L. provided Figure 1 and Figure 5–8. Q.F. provided Figure 2–4. C.L. and C.P. wrote the paper. All authors contributed to discussions of the results. All authors reviewed the manuscript.

## Figures and Tables

**Figure 2:**
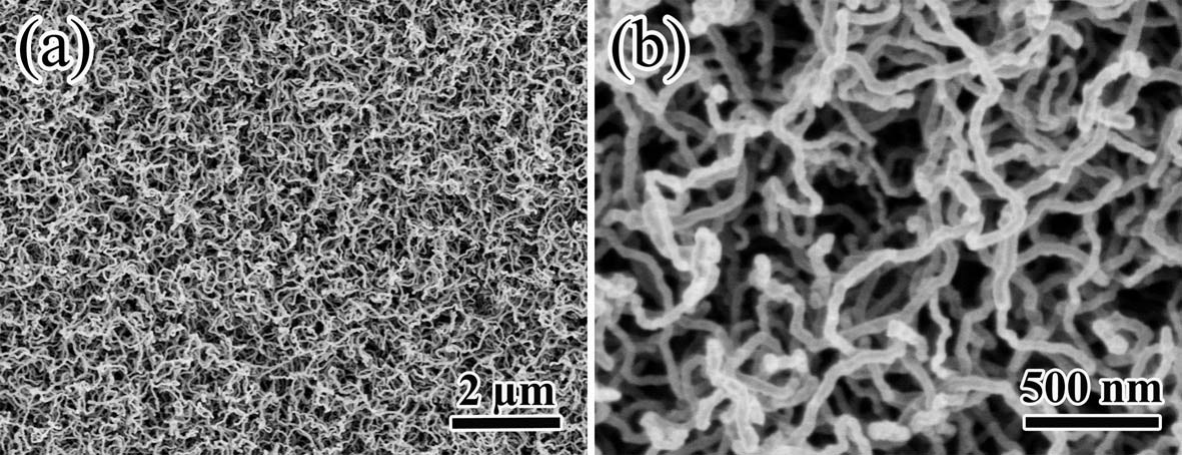
SEM morphologies of CNFs without magnetic field. (a) low magnification, (b) high magnification.

**Figure 3:**
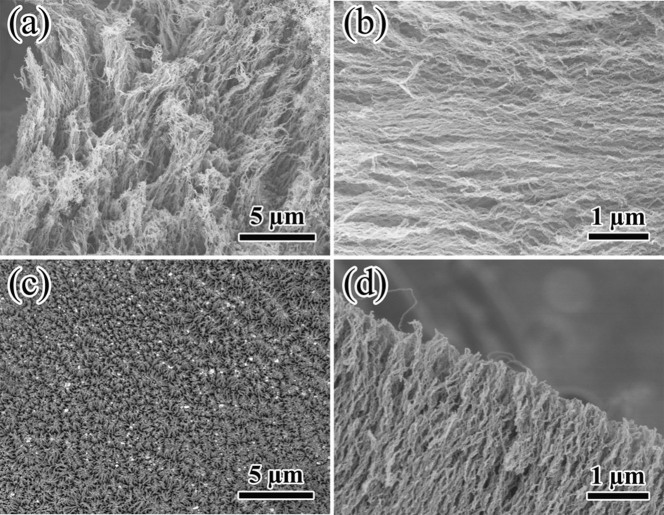
SEM morphologies of CNFs with different magnetic field strengthes. (a) 0.1 T (top view), (b) 0.1 T (side view); (c) 0.5 T (top view), (d) 0.5 T (side view).

**Figure 4:**
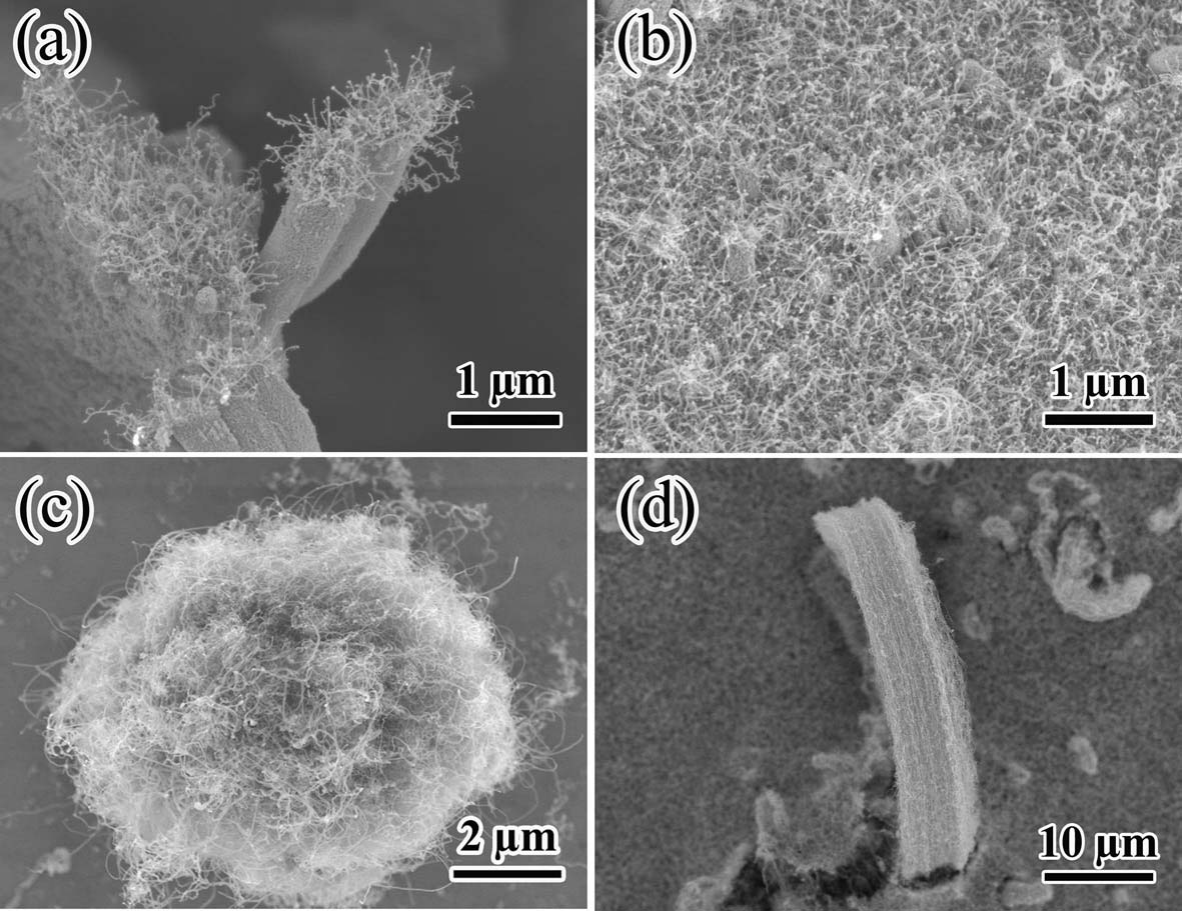
SEM morphologies of CNFs prepared with different parameters. (a) side view and (b) top view of the CNFs prepared with magnetic field 0.5 T for the first 5 min and without magnetic field for the next 5 min; (c) a bundle of CNFs prepared without magnetic field, (d) a bundle of CNFs prepared with the magnetic field line parallel to the substrate.

**Figure 5:**
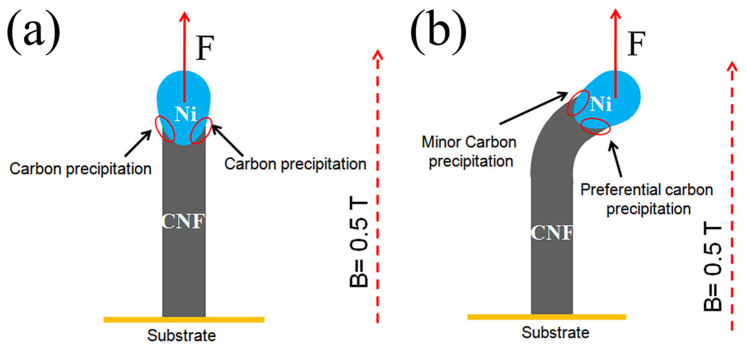
Schematic diagram of alignment mechanism of CNFs in a strong magnetic field. (a) If a CNF grows vertically along the magnetic field line, the gradient of magnetic field creates a repulsive force that carbon atoms will be repelled and precipitate uniformly on both side of the bottom of the catalyst particle/nanofiber interface, which makes CNF continues to grow vertically. (b) If CNF starts to bend due to spatial fluctuations in carbon precipitation at the particle/nanofiber interface, the carbon atoms will still be repelled by the repulsive force and precipitate preferential on the bottom side of the catalyst particle/nanofiber interface. Preferential carbon precipitation leads to the bottom side of the catalyst particle/nanofiber grow faster, which also results in the CNF growth vertically along the magnetic field line.

**Figure 6:**
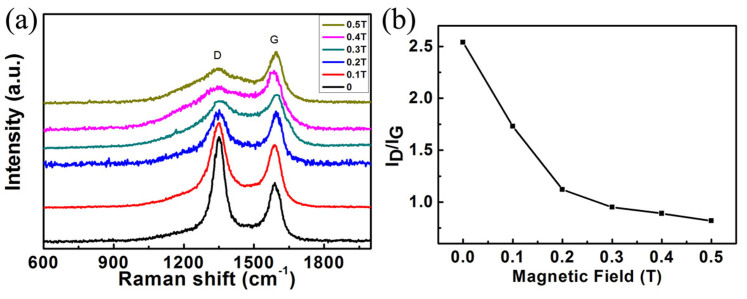
Raman spectra of CNFs prepared with different magnetic field strengthes. (a) Raman spectra, (b) integrated intensity ratio I_D_/I_G_.

**Figure 7:**
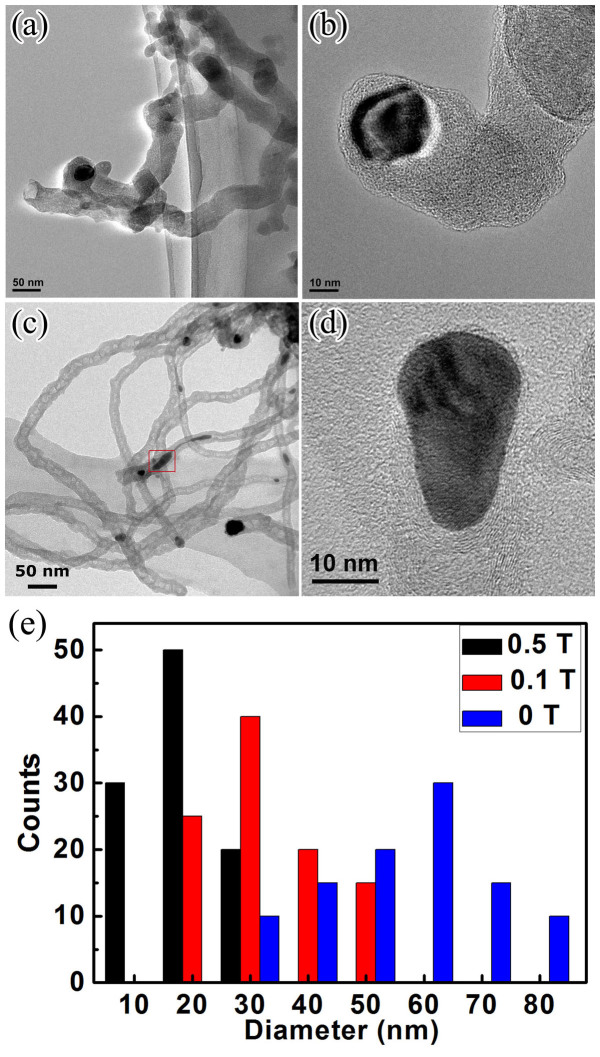
HRTEM micrographs of CNFs. (a) low magnification and (b) high magnification of CNFs prepared without magnetic field; (c) low magnification and (d) high magnification of CNFs prepared with 0.5 T magnetic field; (e) the catalyst particle diameter distribution of the CNFs prepared with different magnetic field strengthes.

**Figure 8:**
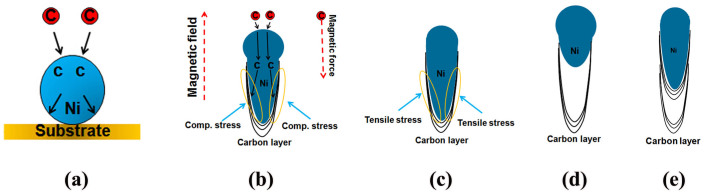
Schematic representation of the growth process of the ordered bamboo-liked CNT with a strong magnetic field (see text).
